# Single‐fraction spine SBRT end‐to‐end testing on TomoTherapy, Vero, TrueBeam, and CyberKnife treatment platforms using a novel anthropomorphic phantom

**DOI:** 10.1120/jacmp.v16i1.5120

**Published:** 2015-01-08

**Authors:** John J. Gallo, Isaac Kaufman, Rachel Powell, Shalini Pandya, Archana Somnay, Todd Bossenberger, Ezequiel Ramirez, Robert Reynolds, Timothy Solberg, Jay Burmeister

**Affiliations:** ^1^ Cancer Care of Maine Radiation Oncology Eastern Maine Medical Center Brewer ME; ^2^ Department of Radiation Oncology McLaren/Northern Michigan Hospital Petoskey MI; ^3^ Webber Cancer Center St. John Providence Health System Warren MI; ^4^ Department of Radiation Oncology Oakwood Healthcare System Dearborn MI; ^5^ Department of Radiation Oncology Huron Valley‐Sinai Hospital Commerce Township MI; ^6^ Department of Radiation Oncology Wayne State University School of Medicine Detroit MI; ^7^ Gershenson R.O.C. Karmanos Cancer Center Detroit MI; ^8^ Department of Radiation Oncology University of Texas Southwestern Medical Center Dallas TX; ^9^ Department of Radiation Oncology University of Pennsylvania School of Medicine Philadelphia PA USA

**Keywords:** stereotactic body radiotherapy, SBRT, single‐fraction spine, SBRT end‐to‐end testing

## Abstract

Spine SBRT involves the delivery of very high doses of radiation to targets adjacent to the spinal cord and is most commonly delivered in a single fraction. Highly conformal planning and accurate delivery of such plans is imperative for successful treatment without catastrophic adverse effects. End–to‐end testing is an important practice for evaluating the entire treatment process from simulation through treatment delivery. We performed end‐to‐end testing for a set of representative spine targets planned and delivered using four different treatment planning systems (TPSs) and delivery systems to evaluate the various capabilities of each. An anthropomorphic E2E SBRT phantom was simulated and treated on each system to evaluate agreement between measured and calculated doses. The phantom accepts ion chambers in the thoracic region and radiochromic film in the lumbar region. Four representative targets were developed within each region (thoracic and lumbar) to represent different presentations of spinal metastases and planned according to RTOG 0631 constraints. Plans were created using the TomoTherapy TPS for delivery using the Hi·Art system, the iPlan TPS for delivery using the Vero system, the Eclipse TPS for delivery using the TrueBeam system in both flattened and flattening filter free (FFF), and the MultiPlan TPS for delivery using the CyberKnife system. Delivered doses were measured using a 0.007 cm^3^ ion chamber in the thoracic region and EBT3 GAFCHROMIC film in the lumbar region. Films were scanned and analyzed using an Epson Expression 10000XL flatbed scanner in conjunction with FilmQAPro2013. All treatment platforms met all dose constraints required by RTOG 0631. Ion chamber measurements in the thoracic targets delivered an overall average difference of 1.5%. Specifically, measurements agreed with the TPS to within 2.2%, 3.2%, 1.4%, 3.1%, and 3.0% for all three measureable cases on TomoTherapy, Vero, TrueBeam (FFF), TrueBeam (flattened), and CyberKnife, respectively. Film measurements for the lumbar targets resulted in average global gamma index passing rates of 100% at 3%/3 mm, 96.9% at 2%/2 mm, and 61.8% at 1%/1 mm, with a 10% minimum threshold for all plans on all platforms. Local gamma analysis was also performed with similar results. While gamma passing rates were consistently accurate across all platforms through 2%/2 mm, treatment beam‐on delivery times varied greatly between each platform with TrueBeam FFF being shortest, averaging 4.4 min, TrueBeam using flattened beam at 9.5 min, TomoTherapy at 30.5 min, Vero at 19 min, and CyberKnife at 46.0 min. In spite of the complexity of the representative targets and their proximity to the spinal cord, all treatment platforms were able to create plans meeting all RTOG 0631 dose constraints and produced exceptional agreement between calculated and measured doses. However, there were differences in the plan characteristics and significant differences in the beam‐on delivery time between platforms. Thus, clinical judgment is required for each particular case to determine most appropriate treatment planning/delivery platform.

PACS number: 87.53.Ly

## I. INTRODUCTION

Stereotactic body radiotherapy (SBRT) is defined as highly precise radiotherapy delivered in 5 or fewer fractions using large doses (∼6−30 Gy) of highly localized and conformal radiation with steep dose gradients around an extracranial target.^1^, ^2^ Single‐fraction spine SBRT involves very high doses in a small treatment volume adjacent to the spinal cord. Treating spine metastasis with SBRT has both excellent pain palliation and local tumor control,^3^, ^4^, ^5^, ^6^ but highly conformal planning and accurate delivery of such plans is imperative for successful treatment without catastrophic adverse effects. QUANTEC (American Association of Physicists in Medicine (AAPM)) reports that myelopathy from stereotactic radiosurgery to spinal lesions appears rare (<1%) when the maximum spinal cord dose is limited to the equivalent of 13 Gy in a single fraction or 20 Gy in 3 fractions.^(7)^ The use of SBRT continues to rise, as a 2011 survey found that 63.9% of radiation oncologists report using SBRT for selected patients.^(8)^ With growing implementation of SBRT treatments which rely on a small number of highly localized treatment fractions, end–to‐end testing is an important practice for evaluating the entire treatment process from simulation through treatment delivery, most importantly evaluating the final dosimetric spatial integrity of delivered dose to a patient. We performed end‐to‐end testing for a set of representative spine SBRT targets planned and delivered using different treatment planning systems (TPSs) and delivery systems in an effort to determine the accuracy of these systems and the process required for their use for SBRT.

## II. MATERIALS AND METHODS

### A. Phantom

**Figure 1:**
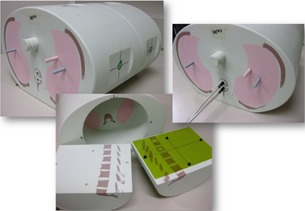
E2E SBRT phantom.

All treatments were planned and delivered using the E2E SBRT phantom (model 036) (Integrated Medical Technologies, Troy, NY), consisting of a thorax body containing specific anthropomorphic internal anatomy including articulated spine, ribs, and lungs. Combined with abdominal phantom (Model 062QA‐35) (IMT, Troy, NY), a novel anthropomorphic phantom ideal for end‐to‐end SBRT testing can be constructed, accepting ion chambers and radiochromic film, which mimics a patient from simulation through treatment, to evaluate measured versus calculated doses (Fig. 1).

### B. Treatment planning

**Figure 2:**
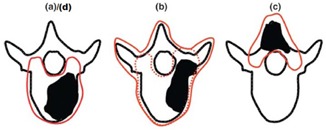
RTOG 0631 target volumes: (a) = Plan A, (b) = Plan B, (c) = Plan C, (d) = Plan D.

All aspects of the treatment planning and delivery processes were performed by radiation oncologists, dosimetrists, medical physicists, and radiation therapists, to best approach the actual representation of a patient's treatment. CT simulation of the phantom was done on a 64 slice Siemens Sensation Open CT simulator (Siemens, Malvern, PA) using an axial acquisition with 1.5 mm slice thickness, 120 kVp, 200 mAs, 120 mA, 50 cm field of view, and 512×512 image matrix, which resulted in a voxel size of 0.977×0.977×1.5 mm. BBs were place on both the thoracic and lumbar isocenters for localizing during planning. Following simulation, four mock planning target volumes (PTVs) were contoured in both the thoracic and lumbar regions using representative target volumes supplied by RTOG 0631^(9)^ as the vertebral body (Plan A), all elements of a single vertebral level completely encircling the spinal cord (Plan B), the posterior spinous process (Plan C), and two consecutive vertebral bodies (Plan D) (Fig. 2). RTOG 0631 also provides an alternative easier PTV for Plan B if an institution were not able to accomplish targeting the complete vertebral segment (red dotted line). This study use the PTVs defined by the solid red line.

A single‐fraction SBRT treatment plan was then developed for delivery using TomoTherapy (Accuray, Sunnyvale, CA), Vero (BrainLAB, Feldkirchen, Germany), TrueBeam (Varian Medical Systems, Palo Alto, CA), and CyberKnife (Accuray, Sunnyvale, CA). All plans had a prescribed dose of 16 Gy to 90% of the target volume, with the following dose constraints for the spinal cord: ≤10 Gy to 0.35 cc of spinal cord and ≤10 Gy to 10% of the partial spinal cord (5 mm above and below target volume), and a max dose of 14 Gy to less than 0.03 cc of the spinal cord. Contouring was done within the Eclipse TPS (v.8.9, Varian Medical Systems) by a radiation oncologist, at which point the contours were exported. All planning systems used a 2 mm dose grid for dose calculation and 6 MV photon beams. Since the CT voxel's largest dimension is 1.5 mm, it motivates a similarly sized dose grid. A smaller dose grid could be chosen if available, but will lengthen already long calculation times and may not improve dosimetric accuracy. Plans were developed by experienced dosimetrists and/or physicists for the following platforms and planning systems: TomoTherapy in the TomoTherapy Hi· Art TPS (v.4.2.1) using convolution/superposition dose algorithm with two helical intensity‐modulated radiotherapy (IMRT) delivery passes; Vero treatment plans were developed in iPlan TPS (v.4.1.2; BrainLAB, Feldkirchen, Germany) using 13 coplanar IMRT beams using the Monte Carlo dose algorithm in the thoracic region and the pencil beam algorithm in the lumbar region; TrueBeam (with the Millennium 120 MLC) treatment plans were developed in Eclipse TPS (v.8.9, Varian Medical Systems) using two full 360° arcs of RapidArc volumetric modulated radiotherapy (VMAT) using the analytical anisotropic algorithm (AAA) in both flattened and flattening filter‐free (FFF) mode; and CyberKnife treatment plans were developed in MultiPlan TPS (v.4.6, Accuray) using three fixed sized collimator cones (10, 15, 25 mm diameter aperture) per plan using the Ray Tracing Algorithm and inverse isocentric planning, with the number of robot node positions from 5672 and the number of beams from 124160.

### C. Plan verification

Each treatment system's respective onboard imaging modality was used for IGRT setup prior to treatment delivery. Specifically, these included MVCT with 3D match for TomoTherapy, CBCT with 3D match for Vero and TrueBeam, and stereoscopic X‐ray with 3D match on CyberKnife. The reader is referred to AAPM Task Group 75^(10)^ for further in‐depth overview of onboard imaging modalities.

The thoracic portion of the E2E SBRT phantom has holes drilled for ion chamber dosimetry, while the lumbar portion has a section designed for film dosimetry. Dose for delivery verification in the thorax was measured using an Exradin A16 0.007 cm^3^ ion chamber (Standard Imaging, Middleton, WI) in the thoracic region placed in both the vertebral body and the spinal cord. In the lumbar region, GAFCHROMIC EBT3 film (Ashland Advanced Materials, Wayne, NJ) was used due to its suggested dose range and ease of use.^11^, ^12^ The film is laser cut to fit reproducibly on pegs for mounting within the phantom.

Ion chamber measurements were single measurements and not average values of multiple deliveries of the same plan, due to the already length delivery times of many of the platforms. These measurements were then compared to the chamber volume average dose from their respective TPS.

Film was digitized using an Epson Expression 10000XL flatbed scanner in conjunction with FilmQAPro2013 (v.3.0.4, Ashland Advanced Materials) for analysis. A calibration curve fit with a rational function

(1) X(D)=a+b/(D−c)

where *X(D)* represents the response at dose D, and *a*, *b*, and *c* are constants, using triple channel dosimetry in a one‐scan protocol developed by Lewis et al.^(12)^ Scanning was performed in transmission mode at 48 bit RGB (16 bit per channel) and 150 dpi resolution, to best approach the resolution of the exported dose maps without undersampling, and saved in tiff file format. The scanner is capable of resolution up to 2400 dpi. Two‐dimensional dose map planes at 102 dpi (corresponding to $0.25\times 0.25\;{\text{mm}}^{2}$ per pixel) were obtained using FilmQAPro from the full 3D dose map exported by each treatment planning system. As resolution is increased, gamma analysis will give better results, particularly in high dose gradient regions^13^, ^14^ allowing use of more stringent analysis constraints.

Further investigation with GAFCHROMIC MD‐V3 film, which has a dose range of 1–100 Gy,^(15)^ was performed as a secondary confirmation of EBT3 dose measurement and for its potential use in SBRT treatment QA. Intuitively one would use a higher dose range film for SBRT QA because, if a 10% minimum threshold is adopted for analysis, the capabilities of the film to measure the low doses is removed, leaving only the higher doses available for analysis. We investigated MD‐V3 with one case on TomoTherapy with Plan B.

Developing and strictly adhering to a scanning protocol within a clinic will ensure reproducibility. As reported in the literature,^12^, ^16^, ^17^, ^18^, ^19^ placement of the film on the scanner bed is very important and should be kept consistent to minimize uncertainty from the nonuniformity of the scanner response. The lateral scan effect on a flat‐bed scanner is responsible for lower pixel values being measured by the scanner the further that measurement is from the central axis of the scanning surface.^12^, ^19^ To keep uncertainties as low as possible, measurements were taken with a small exposed piece of film across both axes of the scanner to determine it's “sweet spot” of uniform response, in which location the treatment film was placed during digitization using the one‐scan protocol.^(12)^ To eliminate film curl, a large piece of clear cut glass was used to completely cover the film during scanning. This compressed the film, eliminating any curl and providing a more uniform measurement. Glass was used when scanning all films, both treatment and calibration films, to account for any additional light attenuation caused by the glass.

A calibration curve was constructed by irradiating films with known doses of 0, 1, 3, 5, 10, 13, 17, and 19 Gy. It also should be noted that not all treatment platforms lend themselves to easy irradiation of a reference film (i.e., TomoTherapy and CyberKnife). Thus, these reference films, along with our calibration curve, were performed on our Varian IX linear accelerator. Calibrating the film dosimetry system in this manner should not be an issue, since EBT3 film has been found to have very weak energy dependence in high energy photon beams^16^, ^20^ and has very weak dose rate dependence^(16)^ provided the linear scaling is done within the same lot of film.^(12)^ If the one scan protocol were not used, a much longer period of time should be waited before analysis of the film. That would allow the dose accumulated on the film to stabilize, as shown by Casanova Borca et al.^(16)^

For analysis, a ROI encompassing the region of film within the fiducial peg holes was used. Gamma analysis criteria of 3%/3 mm, 2%/2 mm, and 1%/1 mm dose/distance‐to‐agreement with a 10% threshold were adopted to evaluate film measurements with calculated 2D dose maps obtained using FilmQAPro from the TPS exported 3D dose maps.^12^, ^13^, ^18^ FilmQAPro allows the user to choose either calculation of gamma using dose criteria relative to the global maximum dose or dose criteria relative to the dose at the specific pixel under analysis. Low and Dempsey's^(13)^ evaluation of the gamma analysis method utilized dose criteria relative to the global maximum dose method. We chose to perform the analysis using both methods, since the former appears to be used more often in the literature and clinic, while the latter represents a more stringent criterion. In addition to dosimetric accuracy, delivery times for each plan on each modality were recorded. While delivery time is not necessarily a concern during end‐to‐ end testing on a phantom, with an actual patient in a clinical situation, length of delivery can be a factor in deciding which modality to utilize.

## III. RESULTS

### A. Thoracic ion chamber region

All platforms and treatment planning systems were able to meet the prescription dose and dose constraints required by RTOG 0631. While there were substantial differences in plan characteristics between platforms which could potentially be clinically significant, the focus of this work is on the validation of delivery accuracy of these plans and the planning results are presented elsewhere.^(21)^ For Plans A, B, and D, ion chamber measurements for all treatment platforms were within 3.2% of the calculated TPS dose (Table 1).

For Plan C, the ion chamber was not within the target volume of the posterior spinous process, as can be seen in the ion chamber placement in Fig. 1, and thus the results for Plan C are not meaningful given the relative uncertainties in such measurements in a very high gradient region. As a result, they are not presented here.

**Table 1:** Thoracic ion chamber measurements in vertebral body

*Treatment Plan*	*Ion Chamber Measurement (Gy)*	*Treatment Platform*				
		*TomoTherapy*	*Vero*	*TrueBeam (Flattened)*	*TrueBeam (FFF)*	*CyberKnife*
Plan A	Calculated Dose	16.5	20.6	16.5	16.3	17.9
	Measured Dose	16.4	21.1	17.0	16.5	18.5
	% Difference	‐0.3	2.6	3.1	1.4	3.0
Plan B	Calculated Dose	16.6	20.8	16.4	16.5	21.8
	Measured Dose	16.6	21.5	16.3	16.6	22.4
	% Difference	0.0	3.2	‐0.3	0.6	2.4
Plan D	Calculated Dose	16.2	21.2	16.0	16.2	20.4
	Measured Dose	16.5	21.5	16.1	16.3	20.6
	% Difference	2.2	1.4	0.6	0.8	0.7

### B. Lumbar film region

During film analysis, only the green channel was considered when performing the gamma analysis because it is suggested using the red channel for 0–10 Gy and green channel for up to 40 Gy for GAFCHROMIC EBT3 film.^11^, ^12^ As can be observed in Tables 2 & 3, gamma analysis of EBT3 film in the lumbar region was exceptional on all platforms using analysis metrics of 3%/3 mm and 2%/2 mm, with a 10% threshold, using percent dose relative to either the global maximum dose or to the local dose.

**Table 2:** Lumbar measurements with GAFCHROMIC EBT3 film with gamma analysis utilizing the dose criterion relative to the global maximum dose method

*Treatment Plan*	*Global* γ*‐analysis constraints w/ 10% threshold*	*Treatment Platform*				
		*TomoTherapy*	*Vero*	*TrueBeam (Flattened)*	*TrueBeam (FFF)*	*CyberKnife*
Plan A	3%/3 mm	100.0	100.0	100.0	100.0	100.0
	2%/2 mm	100.0	99.7	97.9	100.0	100.0
	1%/1 mm	81.3	61.8	77.6	84.3	95.4
Plan B	3%/3 mm	100.0	100.0	100.0	100.0	100.0
	2%/2 mm	99.9	98.7	98.5	99.3	99.3
	1%/1 mm	93.9	62.3	81.5	87.3	85.3
Plan C	3%/3 mm	100.0	100.0	100.0	100.0	100.0
	2%/2 mm	99.9	98.7	98.5	98.9	99.8
	1%/1 mm	93.0	65.2	86.3	85.0	75.4
Plan D	3%/3 mm	100.0	100.0	100.0	100.0	100.0
	2%/2 mm	99.7	100.0	96.9	99.8	100.0
	1%1 mm	87.3	87.5	89.1	90.5	90.4

**Table 3:** Lumbar measurements with GAFCHROMIC EBT3 film with gamma analysis utilizing the dose criterion relative to the pixel under analysis method

*Treatment Plan*	*Local* γ*‐analysis constraints w/ 10% threshold*	*Treatment Platform*				
		*TomoTherapy*	*Vero*	*TrueBeam (Flattened)*	*TrueBeam (FFF)*	*CyberKnife*
Plan A	3%/3 mm	100.0	99.9	100.0	100.0	99.5
	2%/2 mm	98.7	98.3	96.3	99.5	98.9
	1%/1 mm	71.7	49.8	64.7	72.8	84.2
Plan B	3%/3 mm	100.0	99.9	100.0	100.0	96.1
	2%/2 mm	99.9	96.0	97.8	98.9	93.3
	1%/1 mm	85.5	51.3	71.7	79.7	74.3
Plan C	3%/3 mm	100.0	100.0	100.0	100.0	99.2
	2%/2 mm	99.5	100.0	100.0	100.0	97.5
	1%/1 mm	77.1	47.2	81.3	79.2	53.2
Plan D	3%/3 mm	99.5	100.0	100.0	100.0	97.5
	2%/2 mm	96.7	99.8	96.2	99.6	95.1
	1%1 mm	80.7	75.5	81.8	82.1	79.6

### C. Beam‐on delivery time

Treatment beam‐on delivery times vary the most across the different platforms (Table 4). The shortest delivery times are attributed to TrueBeam using FFF, averaging 4.4 min, while the longest delivery times were observed on the CyberKnife, averaging 46.0 min. TrueBeam (flattened), Vero, and TomoTherapy had delivery times between these two extremes, in that order. It should be noted that these cases were also planned for TomoTherapy using the “TomoEDGE” dynamic jaw system and treatment times decreased dramatically to an average delivery time of 6.0 min. The static jaw plans were used for this study since we are not able to deliver dynamic jaw plans on our treatment unit. Similarly, treatment times for CyberKnife represent delivery of plans using three fixed cones. If the iris collimator, or the now‐available MLC attachment, had been used, treatment times would have been shorter.

**Table 4:** Treatment beam‐on delivery times for various treatment platforms and target regions

*Treatment Machine*	*Treatment Region*	*Plan & Delivery Time (min)*				
		*A*	*B*	*C*	*D*	*Average*
TomoTherapy	Thorax	21.1	35.3	28.8	35.3	30.1
	Lumbar	24.8	20.6	27.9	40.8	28.5
				Total Average Delivery Time:		29 min 19 sec
Vero	Thorax	15.0	19.5	21.1	17.2	18.2
	Lumbar	16.8	24.5	21.1	17.2	19.9
				Total Average Delivery Time:		19 min 2 sec
TrueBeam (Flattened)	Thorax	9.6	11.1	7.6	10.2	9.6
	Lumbar	11.2	9.5	7.2	9.5	9.3
				Total Average Delivery Time:		9 min 30 sec
TrueBeam (FFF)	Thorax	4.3	5.5	3.5	4.1	4.3
	Lumbar	4.9	4.6	3.7	4.8	4.5
				Total Average Delivery Time:		4 min 24 sec
CyberKnife	Thorax	50.0	44.4	44.5	46.0	46.2
	Lumbar	43.9	46.9	40.8	42.6	43.5
				Total Average Delivery Time:		45 min 48 sec

## IV. DISCUSSION

The IMT anthropomorphic phantom used in this study provides the capabilities of multiple dose measurement tools, while at the same time providing an excellent representation of an actual patient's anatomy and thereby facilitating localization based on image guidance. In turn, we are able to meet recommendations by TG101 to perform end‐to‐end tests that integrate image guidance and delivery.^(1)^ When performing treatment verification of high dose, highly conformal, and potentially time consuming SBRT treatments, concerns of accurate dose measurement immediately arise. For ion chamber measurements performed over an extended period of time, leakage current can become an immediate concern if not kept very minimal. Leakage was evaluated in this study, with the highest leakage current reaching 0.004 pA, which accounts for less than 0.6% of the measured dose in the longest treatment plan. Thus, leakage contribution is considered to be negligible.

Imaging dose is also a concern. While there is a dose delivered from the localization image‐guided techniques, it has been shown that the dose from this imaging is insignificant in comparison to the dose received from the actual treatment, in particular when treating with single fraction SBRT.^(10)^ In the case of kV‐CBCT, the typical dose delivered is approximately 1.6–2.3 cGy,^10^, ^22^ which is only 0.14% of the prescription dose. Similarly, for MVCT the typical dose is approximately 1.0–3.0 cGy,^10^, ^23^ which represents less than 0.20% of the prescription dose. On the CyberKnife system, the typical imaging dose is on the order of 0.25‐0.50 mGy per image per stereoscopic X‐ray pair.^(10)^ Typically three to four images were needed for CyberKnife setup in this study, totaling the imaging dose to 0.1–0.2 cGy, approximately 0.013% of the prescription dose. The dose contribution of these image‐guidance techniques to the total dose to the film is therefore insignificant, particularly given that a 10% minimum threshold was used in the gamma analysis, which completely removes that region of imaging dose contribution along with any low dose background noise.

**Figure 3:**
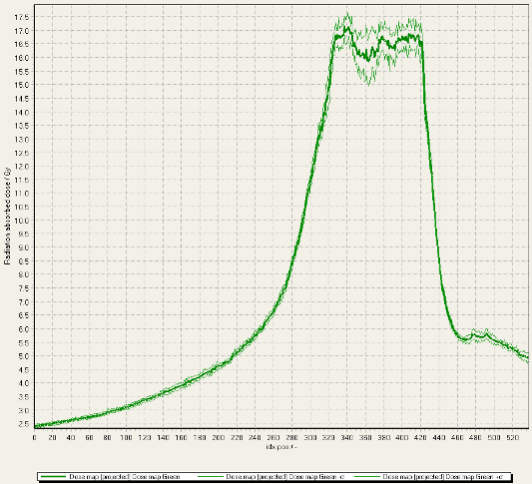
Cross profile of Plan A delivered by TrueBeam with a flattened beam showing the FilmQAPro software calculated uncertainty of the measured dose. The software itself builds a sigma value based upon the scan of the film and the value that is reported for gamma analysis is the middle profile.

The choice of GAFCHROMIC EBT3 film was due in part to its ability to accurately measure the expected dose range,^11^, ^12^, ^18^, ^24^ allowing use up to 40 Gy with the green channel.^11^, ^12^ While EBT2 has a similar composition and thickness of the sensitive layer as EBT3, the most important change is the symmetric layer configuration of EBT3 to eliminate side orientation dependence, which has been reported for EBT2 film.^(25)^ However, a very important dependence of landscape versus portrait orientation of the film during scanning is still prevalent with EBT3.^12^, ^25^ Triple‐channel dosimetry with two point linear dose scaling was used as part of the FilmQAPro2013 software to calibrate treatment films, as described by Lewis et al.^(12)^ The software also provides tools for uncertainty analysis of the film. It can be seen in Fig. 3 that σ≈±3%, which equates to about 0.5 Gy uncertainty within the prescribed target volume.

FilmQAPro also allows for the use of additional reference films if more accurate rescaling is desired. While this could be advantageous for analysis of SBRT treatment films because of the large dose range needing to be scaled, it was not pursued as part of this investigation.

**Figure 4:**
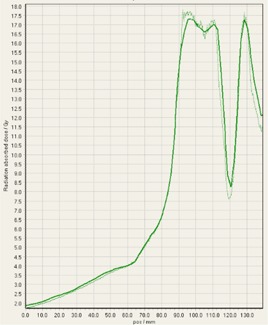
EBT3 film cross profiles vs. TPS dose maps analyzed in FilmQAPro2013 of target volume B delivered on TomoTherapy.

**Figure 5:**
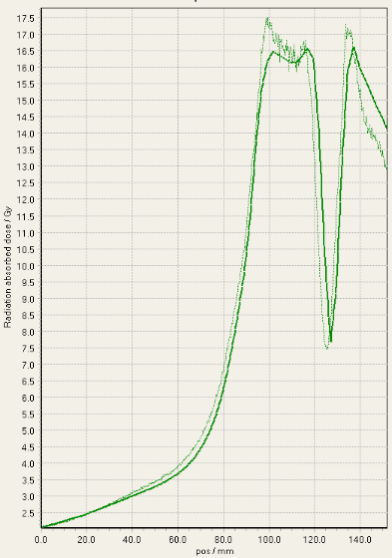
EBT3 film cross profiles vs. TPS dose maps analyzed in FilmQAPro2013 of target volume B delivered on TrueBeam (flattened beam).

**Figure 6:**
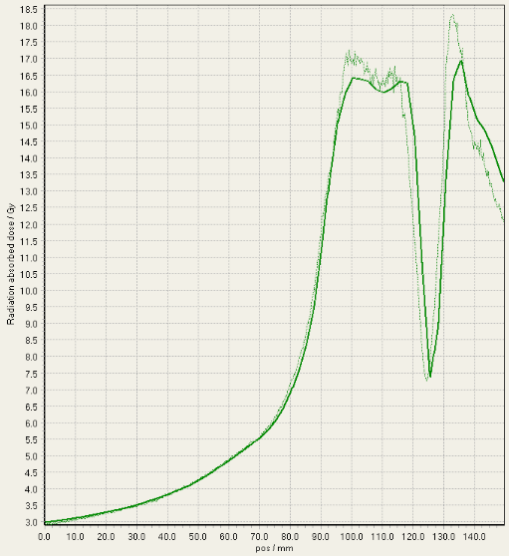
EBT3 film cross profiles vs. TPS dose maps analyzed in FilmQAPro2013 of target volume B delivered on TrueBeam (FFF beam).

**Figure 7:**
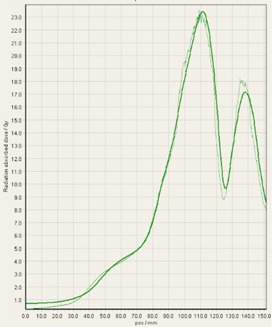
EBT3 film cross profiles vs. TPS dose maps analyzed in FilmQAPro2013 of target volume B delivered on CyberKnife.

**Figure 8:**
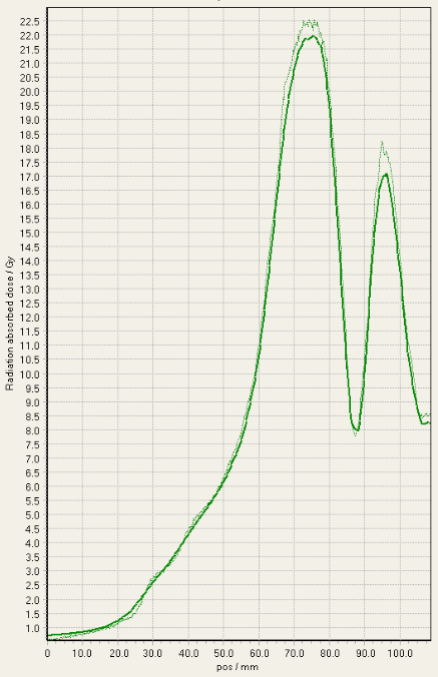
EBT3 film cross profiles vs. TPS dose maps analyzed in FilmQAPro2013 of target volume B delivered on Vero.

During analysis of each film, it was interesting to observe that some of the treatment platforms, while still having exceptional gamma passing rates, delivered slightly higher doses within the target volume in comparison to TPS predictions, and either matched or gave lower doses to normal tissue regions outside of the target volume (Figs. 4–8). This slight overdosing, that seemed to occur within the high‐dose target volume, was in accordance with the higher doses measured with the ion chamber measurements within the target volumes of the thoracic region. In addition, in order to meet the desired RTOG 0631‐prescribed dose constraints of at least 16 Gy to at least 90% of the target, the average dose within the target volume was increased substantially, as indicated in the planned doses of the ion chamber measurements (Table 1). This is particularly relevant for Vero and CyberKnife, for which the maximum doses within the target volume reached to 18–23 Gy. This is 12.5%–43.7% more than the prescription dose of 16 Gy. While goal of these plans was not dose homogeneity (RTOG allows dose inhomogeneity existing within the target volume), TG101 supports the idea that hot spots confined within the target are generally clinically desirable, and may offer an advantage in eradicating radio‐resistant hypoxic cells.^(1)^ It was considered that the slightly higher dose may also be a result of reaching a limit of the usable dose curve for EBT3 film or a limit of the Epson Expression 10000XL flatbed scanner as the measurement in these high‐dose target regions also are slightly noisy. However, Casanova Borca et al.^(16)^ showed that EBT3 film should work up to nearly 40 Gy without a problem.

As also can be seen by referring to the dose profiles and Fig. 1 of the phantom, depending on the selected target, the discrete size and the locations of the localizing holding pegs of the film limits extension of the dose profile much past the posterior spinous process and either approaches the edge of the phantom itself or runs into a peg. In addition, the numerical units of the x‐axis “position” on the profiles are simply arbitrary to where the ROI in FilmQAPro was placed, and due to slight variations, the graph grid size auto adjusted to the profiles presented here.

Our brief investigation shows the MD‐V3 provides similar gamma passing results as EBT3 film (Table 5). Thus, SBRT QA using radiochromic film does not warrant the use of the more expensive MD‐V3 film, but does reaffirm that the high‐dose measurements with EBT3 were producing an appropriate response.

With total treatment times that can extend over an hour, patient comfort is an important aspect of SBRT. Knowing that cases of spine metastasis typically involve various levels of pain, a patient may not want — or be able — to undergo a long treatment, even if that means they would receive a slightly better treatment. While all of the treatments that were delivered on the platforms described here utilize image guidance that allows for interfraction realignment, there is still no guarantee that the patient will not move after those adjustments have been performed. Additionally, the delivery times provided reflect only “beam‐on” delivery time. Setup and imaging time will be an addition to the recorded times. This additional time could be estimated, but will fluctuate significantly from clinic to clinic based upon the mobility/cooperation of the patient, the skill of the therapists setting up the patient in the SBRT immobilization devices, the skill of the therapist navigating the IGRT functionalities of the treatment platform, and the efficiency of real‐time physician approval of the setup and image guidance. Secondly, observations by Wang et al.^(26)^ and Morgan et al.^(27)^ suggest that the total time to deliver a single fraction may have a significant impact on treatment outcome for tumors, reducing its biological effectiveness. Further investigation is needed in relation to SBRT, as these studies investigated the effect of delivery time on radiobiological effectiveness in relation to intensity‐modulated radiation therapy (IMRT) delivery. In accordance, Benedict et al.^(28)^ showed similar results in the loss of biological effectiveness for human glioma cells *in vitro* with high stereotactic radiotherapy (SRT) doses as the irradiation time was increased.

**Table 5:** Comparison of EBT3 vs. MD‐V3 GAFCHROMIC film for gamma analysis of TomoTherapy plan B using both dose criteria relative to maximum and also to the pixel in question

*Treatment Plan*	γ*‐analysis constraints w/ 10% threshold*	*GAFCHROMIC Film*	
		*EBT3*	*MD‐V3*
TomoTherapy Plan B	3%/3 mm	100 / 100	100 / 100
	2%/2 mm	100 / 99.9	100 / 99.9
	1%/1 mm	97.4 / 91.9	98.9 / 84.5

## V. CONCLUSIONS

End‐to‐end testing of SBRT treatment platforms is an important step in assessing the quality of an institution's SBRT capabilities. The IMT anthropomorphic phantom combined with ion chamber and GAFCHROMIC EBT3 film measurements is an ideal method for verification of spinal SBRT treatments. While most clinical treatment QA is performed using analytical criteria less stringent than those employed here, commonly using 2%/2 mm as the most stringent criteria, use of higher resolution in both scanned film and calculated dose maps can facilitate constraints of 1%/1 mm for more precise analysis. Gamma analysis criteria such as 3%/1 mm or 2%1 mm can also be adopted as a strong measure of spatial integrity while still allowing for flexibility in the dosimetric accuracy of both the delivery platforms and of the dosimetric uncertainties of the film, although these were not presented here. We have found that the treatment planning systems for all platforms were able to meet the constraints outlined by RTOG 0631, and more importantly, that all treatment platforms were able to deliver those plans extremely accurately within the delivery and mechanical tolerances of the treatment platforms and suggested AAPM task group guidelines.^1^, ^29^ While treatment plan characteristics may vary between platforms and treatment delivery times vary greatly, clinical judgment must be applied to determine the most appropriate treatment planning/delivery platform for each particular case based on all aspects of the treatment plan.
